# Central Airway Recanalisation Using Dual Debulking With Para‐Toluenesulfonamide (CLEAR‐DUAL‐PTS)

**DOI:** 10.1002/rcr2.70285

**Published:** 2025-07-25

**Authors:** Chen Yong Tan, Hui‐Xin Tan, Yen Shen Wong, Sumithra Appava, Shoban Raj Vasudayan, Noriah Othman, Swee Wei Leong, Mona Zaria Nasaruddin, Jamalul Azizi Abdul Rahaman

**Affiliations:** ^1^ Pulmonology Department Hospital Sultan Idris Shah Serdang Malaysia; ^2^ Pathology Department Hospital Sultan Idris Shah Serdang Malaysia

**Keywords:** central airway obstruction, para‐toluenesulfonamide (PTS), pulmonary adenoid cystic carcinoma (PACC)

## Abstract

Pulmonary adenoid cystic carcinoma (PACC) is a rare salivary gland‐type malignancy of the tracheobronchial tree, accounting for 0.04%–0.2% of primary lung cancers. It may cause severe malignant airway obstruction (SMAO), necessitating urgent intervention. Surgical resection remains the primary treatment, but inoperability or patient preference may require alternative strategies. We report a case of a 57‐year‐old non‐smoking female with PACC‐SMAO who presented with total left lung collapse due to left main bronchus (LMB) involvement. She declined surgery and underwent bronchoscopic dual debulking combining electrocautery snaring with repeated intratumoural para‐toluenesulfonamide (PTS) injections. The intervention safely reduced tumour burden, restored airway patency, and significantly improved clinical and radiological outcomes without adverse events. Our case underscores PTS as a promising adjunct therapy for inoperable PACC‐SMAO, warranting further investigation into its long‐term efficacy and optimal dosing. This innovative approach may significantly redefine palliative care options.

## Graphical abstract

We describe a case of pulmonary adenoid cystic carcinoma with severe malignant airway obstruction successfully managed with bronchoscopic dual debulking—mechanical debulking via electrocautery snaring and chemical debulking with intratumoural para‐toluenesulfonamide injection, leading to significant clinical and radiological improvement.

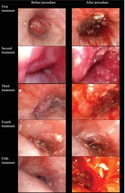

## 1 Introduction

Pulmonary adenoid cystic carcinoma (PACC), historically known as ‘cylindroma’, is a rare salivary gland tumour arising from the tracheobronchial submucosal glands, accounting for only 0.04%–0.2% of all primary lung malignancies tumours [1]. It predominantly affects the 40–60 age group. PACC varies in presentations, from asymptomatic to symptomatic, such as cough, dyspnoea, wheezing, haemoptysis, and obstructive pneumonia. It is often undetectable on standard chest radiograph, rendering computed tomography (CT) essential for assessing tumour location, extent, invasion, and intrapulmonary metastases.

Surgical resection remains the primary therapy for PACC. Endoscopic interventions, radiotherapy, and chemotherapy are options for palliative treatment in patients who are unsuitable or unwilling to undergo surgery. Emerging targeted therapies show promise by inhibiting pathways such as the myeloblastosis (MYB) proto‐oncogene, epidermal growth factor receptor (EGFR) mutation, and vascular endothelial growth factor receptor (VEGFR), while immune checkpoint pathways like programmed death‐ligand 1 (PD‐L1) show low response rates.

Intratumoural therapy offers enhanced bioavailability, remodelling the tumour microenvironment and simultaneously reducing systemic and immune‐related adverse effects [2]. Tumouricidal agents such as ethanol absolute, cisplatin and 5‐fluorouracil have been utilised in lung cancer treatment [3]. Para‐toluenesulfonamide (PTS), a low‐molecular‐weight hydrophobic compound, has shown tumouricidal effects with efficacy verified in breast, liver, head and neck cancers and non‐small cell lung cancer with severe malignant airway obstruction (NSCLC‐SMAO). Intratumoural PTS injection increases forced expiratory volume in 1 s (FEV_1_), enhances aeration and extends median survival. This therapeutic benefit has also been observed in PACC with SMAO (PACC‐SMAO) [4].

We describe a case of PACC‐SMAO successfully managed with bronchoscopic dual debulking—mechanical debulking via electrocautery snaring and chemical debulking with intratumoural PTS injection, leading to significant clinical and radiological improvement.

## 2 Case Report

A 57‐year‐old non‐smoking woman with hypertension, diabetes mellitus, dyslipidaemia and hypothyroidism presented with progressive dyspnea for 1 year. Physical examination revealed diminished breath sounds over the left lung with oxygen desaturation. Chest radiograph revealed near‐total left lung white‐out with tracheal deviation.

**FIGURE 1:**
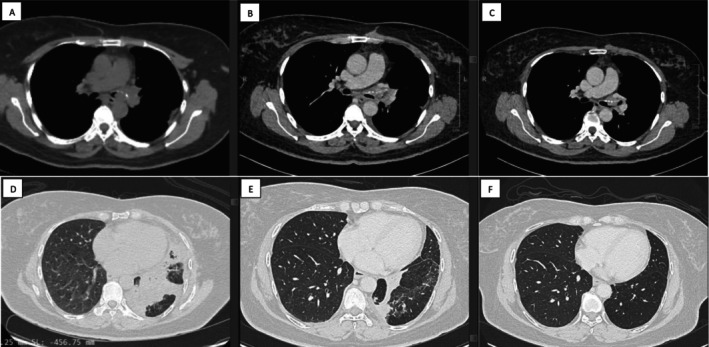
Computed tomography (CT) scan images of the thorax of the case. (A) At diagnosis, the adenoid cystic carcinoma mass (ACC) totally occluded the left main bronchus (LMB). (B) CT scan after two procedures showed some canalisation of the LMB. (C) CT scan after five procedures demonstrated a smaller mass and a linear passage at LMB. (D–F) The collapse consolidation of the left lower lobe improved with the dual debulking treatment from diagnosis, post‐second procedure and post‐five procedures.

**FIGURE 2:**
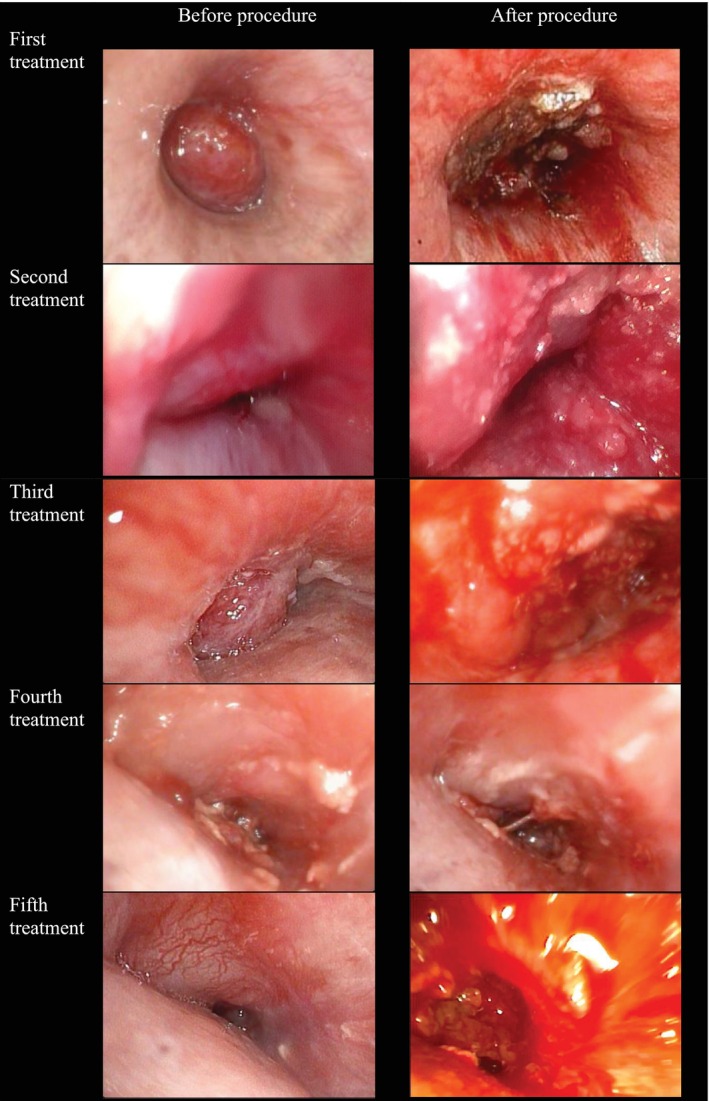
Endobronchial image of treatment log.

CT scan revealed a 0.9 × 2.8 × 2.2 cm ill‐defined hypodense endobronchial lesion within the left main bronchus (LMB), causing complete obliteration (Figure 1). Bronchoscopy confirmed LMB total occlusion by a hypervascular polypoidal mass (Figure 2), and biopsy's histopathological examination (HPE) established adenoid cystic carcinoma (ACC). Positron emission tomography‐computed tomography (PET‐CT) scan showed no distant metastasis, fluorodeoxyglucose (FDG)‐avid focal LMB mass with maximum standardised uptake value (SUVmax) 8.2, staging at IVA (T4N0M0). The tumour board recommended surgical intervention with possible left pneumonectomy, but the patient declined.

She opted for dual debulking (Figure 2):

First session: Electrocautery snaring, cryoextraction and 4 mL PTS (330 mg/mL) were injected at four spots, resulting in noticeable tumour discoloration without bleeding.

Follow‐up bronchoscopy (1 week): Sloughing was observed without active bleeding.

Second session (2 weeks): 4 mL PTS administered, enabling visualisation of the lower lobe bronchus after necrotic tissue removal.

Third session (3 weeks): Additional dual debulking, with argon plasma coagulation (APC) applied to control bleeding. Bronchoscope Pentax EB15‐J10 (outer diameter 5.4 mm) able to pass the tumour and visualise both left upper and lower lobe bronchi.

Fourth session (7 weeks): Endobronchial ultrasound (EBUS)‐guided 2 mL PTS injection into the extraluminal tumour.

Fifth session (11 weeks): Endobronchial injection of 5.25 mL PTS with immediate debridement of necrotic tissue.

The patient experienced no adverse effects, showed symptom improvement after the third injection, and remained symptom‐free despite residual tumour.

## 3 Discussion

This case report highlights intratumoural PTS injection as a novel adjunctive treatment for PACC‐SMAO. Intratumoural PTS injection significantly debulks the tumour, chemically ameliorating airway obstruction.

PACC is a rare salivary gland‐type malignancy causing SMAO as over 90% arise in the central airway [1]. Unlike other lung cancers, PACC has equal gender distribution and a higher prevalence in non‐smokers, typically affecting those aged 40–60, as demonstrated by our case involving a 57‐year‐old non‐smoker [1]. PACC is histopathologically divided into grade I tubular, grade II cribriform and grade III solid tumours. Immunohistochemically, high expression levels of CK7, CD117, P63, SMA, CK5/6 and S‐100 are helpful for the diagnosis. The HPE in our case showed both tubular and cribriform patterns.

Surgery is the preferred therapeutic approach, however factors such as anaesthesia and surgical risks, and patient preferences, play a role in decision‐making. Surgery alone offers higher 5‐ and 10‐year survival rates of 88%–100% and 51%–80%, respectively, compared to definitive radiotherapy of 34.9% and 16.1%, respectively [4]. Notably, the longest survival time in the operable group was reported up to 253 months [1]. Advanced PACC responds poorly to palliative chemotherapy and radiotherapy. EGFR mutations are rare in salivary gland carcinoma, rendering EGFR‐tyrosine kinase inhibitors (EGFR‐TKIs) not the ideal targeted therapy [1]. PACC‐SMAO warrant bronchoscopic intervention, with repeated procedures required due to persistent disease or treatment‐related complications. Prognostic factors influencing the survival include tumour stage, location, positive surgical margin and treatment modality.

PTS efficaciously induces tumour necrosis and can be safely administered over multiple sessions [3]. Studies have reported an 85.7% airway tumour size reduction with a median survival of 4.98 years [5]. In this case, all procedures for the case were performed using rigid bronchoscopy, which provided superior airway control, allowing for greater precision, improved operating conditions, and enhanced protection of instruments. Total debulking was challenging: after removal of the first half of the elongated mass, no identifiable stalk remained. The tumour was extensively infiltrative, extending along the entire left main bronchus up to the bifurcation of the lower lobe, rendering complete debulking unachievable. Mechanical debulking alone without PTS may not have achieved the outcome observed in this case, as repeated PTS injections helped reduce the surrounding tumour burden further. PTS was particularly helpful in the absence of an identifiable stalk for snaring. It provided a means for achieving adequate local tumour control while avoiding airway stenting and its complications [5]. While severe adverse events, including worsened airway obstruction due to swelling and necrotic debris, have been reported [3], post‐procedure effects in our case were mild. No injury to surrounding healthy tissue has been observed, consistent with Lu′s study [4]. Nonetheless, several limitations exist, which include technical challenges such as dosing constraints (maximum 10 mL per session), delivery difficulties due to viscosity, and the need for repeated procedures. Additional barriers include limited availability, the requirement for special approval from local authorities, and cost considerations.

In conclusion, this case highlights PTS intratumoural injection as a novel potential adjunctive treatment for inoperable PACC‐SMAO, demonstrating clinical and radiological improvement without significant adverse events.

## Author Contributions

C.Y.T. conceived the conceptualisation. C.Y.T. and H.‐X.T. wrote the manuscript with support from Y.S.W., S.A., S.R.V. and N.O. S.W.L., M.Z.N. and J.A.A.R. aided and reviewed the manuscript. All the authors discussed and contributed to the final manuscript.

## Ethics Statement

This case report is registered under National Medical Research Register (NMRR) (NMRR ID: NMRR ID‐25‐00059‐ILS) and is waived for ethical approval because it is a case report with anonymous identity.

## Consent

The authors declare that written informed consent was obtained for the publication of this manuscript and accompanying images using the consent form provided by the Journal.

## Conflicts of Interest

The authors declare no conflicts of interest.

## Data Availability

The data that support the findings of this study are available on request from the corresponding author. The data are not publicly available due to privacy or ethical restrictions.
